# Lower levels of α-Klotho in serum are associated with decreased lung function in individuals with interstitial lung abnormalities

**DOI:** 10.1038/s41598-019-47199-0

**Published:** 2019-07-25

**Authors:** Ivette Buendia-Roldan, Nancy Machuca, Mayra Mejía, Mariel Maldonado, Annie Pardo, Moises Selman

**Affiliations:** 1 Instituto Nacional de Enfermedades Respiratorias “Ismael Cosío Villegas”, México city, Mexico; 2 Facultad de Ciencias, Universidad Nacional Autónoma de México, México city, Mexico

**Keywords:** Prognostic markers, Predictive markers, Prognostic markers, Predictive markers

## Abstract

Interstitial lung abnormalities (ILA) represent aging-associated bilateral interstitial abnormalities in nondependent areas of the lung. However, the aging mechanisms associated with ILA remain uncertain. α-Klotho is an anti-aging molecule that decreases progressively with age, and abnormally low circulating levels of this protein have been revealed in several chronic-degenerative diseases. In this study, we evaluated α-Klotho serum concentrations in individuals with ILA, and examined whether its levels were associated with pulmonary function decline. α-Klotho was measured by ELISA in 50 respiratory asymptomatic adults with ILA and 150 healthy individuals over 60 years. Compared with controls, ILA subjects were predominantly older males, and showed lower lung diffusing capacity (DLCO), higher desaturation after exercise, and higher concentrations of serum matrix metalloprotease-7 (6.24 ± 4.1 versus 4.3 ± 1.7 ng/ml; p = 0.002). No differences were found in serum concentrations of α-Klotho. However, lower levels of this protein in ILA significantly correlated with lower values of forced vital capacity (Rho = 0.39; p = 0.005), forced expiratory volume in one second (Rho = 0.39; p = 0.005), and DLCO (Rho = 0.29, p = 0.04). These findings suggest that decreased concentrations of α-Klotho may be a predictive biomarker of accelerated decline of lung function in individuals with ILA.

## Introduction

In the last years, a growing body of evidence indicates that around 8 percent of adults over 60 years, primarily smokers, develops interstitial lung abnormalities (ILA), defined as the presence of different patterns of increased lung density, including ground-glass attenuation and reticular opacities, on chest high resolution computed tomography (HRCT)^1^. ILA are associated with lung functional decline, increased respiratory symptoms, and an increased risk of all-cause and respiratory-specific mortality^2,3^. However, the risk factors, pathogenic mechanisms, and putative consequences remain unclear.

The mammalian *klotho* gene encodes a 130 kDa type I single-pass transmembrane glycoprotein called α-Klotho, which is expressed in various organs, and is implicated in pleiotropic biological functions^4^. The extracellular domain of membrane Klotho consists of two repeat sequences that are released into circulation after cleaved by the ADAM-10 and ADAM-17 (a disintegrin and metalloproteinase domain-containing proteins 10 and 17). Soluble α-Klotho may be detected in the blood, urine, and cerebrospinal fluid, and display several hormonal functions including the inhibition of intracellular insulin and IGF1 signaling^4,5^. The membrane-bound form of Klotho is mainly involved in fibroblast growth factor (FGF) receptor signaling. α-Klotho is considered an aging-suppressor protein derived from the initial observation that the α-Klotho deficient mice exhibited a severe aging-like phenotype including arteriosclerosis, skin atrophy, gonadal dysplasia, infertility, osteoporosis, and emphysema, and an overall shorter life span^6^. Moreover, upregulation of Klotho in transgenic mice significantly extended their lifespan^6^.

Levels of soluble α-Klotho correlate negatively with age, and abnormally low concentrations of the protein have been reported in several aging-associated chronic-degenerative diseases including chronic obstructive pulmonary disease, and idiopathic pulmonary fibrosis^7,8^. Importantly, α-Klotho protects cells from oxidative stress and inhibits several pathways related with lung fibrosis, including Wnt/β-catenin signaling and TGF-β1^9^. Thus, we hypothesized that lower circulating α-Klotho could be associated with the development of interstitial lung abnormalities and/or with the decline of pulmonary function tests. In this context, the aims of the present study were to find out whether α-Klotho serum concentrations distinguish individuals with ILA in an aging population, and to evaluate whether its levels are associated with some pulmonary function tests.

## Results

Demographic and functional characteristics of the studied population are shown in Table 1. Compared with the control group, individuals with ILA were slightly older and with a higher percentage of males. Consistent with previous studies^10^, ILA subjects showed increased serum concentration of matrix metalloprotease-7 (6.24 ± 4.1 ng/ml versus 4.3 ± 1.7 ng/ml; p = 0.002). No differences were found in the reported history of cigarette smoking or in the presence of aging-associated comorbidities.

**Table 1:** Demographic and functional characteristics.

Variable	ILA (n = 50)	Control (n = 150)	p value
Male, n (%)	22 (44)	41 (27)	0.03
Age, years, mean (SD)	73 (±9)	69 (±8)	0.05
BMI, kg/m^2^, mean (SD)	27 (±4)	27 (±4)	0.15
Smoking, n (%)	22 (44)	53 (35)	0.31
MMP-7 serum concentration, mean (SD); ng/ml	6.24 (±4.1)	4.3 (±1.7)	0.002
FEV_1_, L, mean (SD)	2.1 (±0.5)	2.3 (±1.7)	0.38
FEV_1_, % predicted	99 (±16.5)	102 (±20.1)	0.36
FVC, L, mean (SD)	2.7 (±0.7)	2.8 (±0.8)	0.40
FVC, % predicted, mean (SD)	93 (±14.6)	96 (±18.8)	0.20
DL_CO_, % predicted, mean (SD)	96 (±23)	115 (±22.3)	<0.001
SpO_2_ rest, %, mean (SD)	94 (±2.0)	94.2 (±2.2)	0.32
SpO_2_ exercise, %, mean (SD)	87.5 (±7.6)	91.6 (±5.6)	<0.001
6MWT, meters, mean (SD)	404 (±146.1)	439 (±119)	0.09

**BMI:** Body mass Index, **MMP-7**: Matrix metalloprotease-7, **FEV**_**1**_: Forced expiratory volume in 1 second, **FVC:** Forced vital capacity, **DL**_**CO**_**:** Diffusing capacity of the lung for carbon monoxide, **SpO**_**2**_**:** Blood oxygen saturation, **6MWT**: 6-minute walk test. Pulmonary Function Tests were performed at 2440 mts of altitude.

Patients with ILA displayed a mild but significant decrease of functional tests indicators of abnormal gas-exchange. Thus, the mean ± standard deviation of DLCO was 96 ± 23% predicted in ILA subjects versus 115 ± 22% in the control group (p < 0.001). Likewise, individuals with ILA showed a higher desaturation after exercise (SO_2_ 86 ± 5.8% versus 89 ± 5.3; p < 0.001), and a non-significant shorter 6-min walk distance. No differences were observed in FVC and FEV_1_.

We did not find statistically significant differences in the mean serum concentration of α-Klotho between the two groups: ILA: 715 ± 456 pg/ml, controls: 744 ± 438 pg/ml, (p = 0.6).

**Figure 1:**
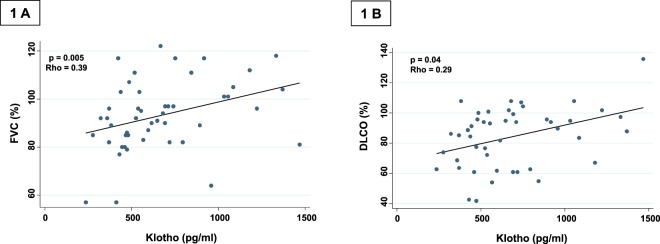
Spearman correlation between α-Klotho serum concentrations and forced vital capacity (FVC) (**A**), and diffusing capacity of the lung for carbon monoxide (DLCO) % predicted (**B**). Inserted lines represent regression line.

However, in the ILA cohort, we found that low levels of α-Klotho serum concentrations significantly correlated with lower values of FVC% predicted (Rho 0.39; p = 0.005, Fig. 1A), FEV1% predicted (Rho 0.39; p = 0.005), and DLCO% predicted (Rho 0.29, p = 0.04, Fig. 1B). Since ILA likely represent an inflammatory response of the lung parenchyma, we examined whether the levels of α-Klotho in patients with ILA correlated with serum concentration of C-reactive protein, a known biomarker of inflammation. We found a significant negative correlation (Rho −0.33; p = 0.02). By contrast, there was no correlation between the serum levels of α-Klotho and the concentrations of MMP-7 (Rho −0.09; p = 0.5).

In the control group, serum α-Klotho concentrations did not correlate with any parameter of lung function tests (FVC (Rho −0.10; p = 0.22), FEV1 (Rho −0.11; p = 0.16), and DLCO (Rho 0.039, p = 0.63). Likewise, no association was observed with the levels of CRP (Rho 0.06; p = 0.45).

Twenty out of the 50 individuals with ILA completed 1 year of follow-up. We determined serum concentration of α-Klotho and made a correlation with pulmonary function tests performed on the same day. A positive correlation was also found for FVC% predicted (Rho 0.55 p = 0.01) and FEV1% predicted (Rho 0.54, p = 0.01). However, no significant correlation was observed with DLCO.

## Discussion

Our findings indicate that in individuals with ILA, the circulating levels of the anti-aging protein α-Klotho did not differ from lung-healthy older individuals but show a positive correlation with lung functional parameters including both the gas exchange and spirometry, suggesting that it may be a pathobiological predictive biomarker of functional decline. In other words, lower levels of Klotho may identify individuals with ILA who are more likely to experience an unfavorable effect on pulmonary function tests than similar individuals with normal levels of Klotho^11^. Pulmonary functional abnormalities has been previously observed in patients with ILA^12^ and a correlation between 6 minute walk test and FVC% predicted has been also reported^3^. However, the mechanisms by which low levels of α-Klotho are associated to this functional decline are presently unknown. Klotho expression is decreased in the lungs of smokers and further reduced in patients with chronic obstructive pulmonary disease (COPD) which is associated with increased oxidative stress, inflammation and apoptosis^7^. Moreover, recently it was shown that cigarette smoke induces airway inflammation by downregulation of Klotho and activation of fibroblast growth factor receptor-4 in the airway epithelium of patients with COPD^13^. Likewise, a recent study showed that circulating Klotho is also decreased in IPF and that Klotho-deficient mice are more susceptible to lung inflammation and fibrosis^8^. Interestingly, we found a negative correlation of α-Klotho and CRP, suggesting a role of inflammation in the development of ILA. Elevations in baseline CRP level have been used to determine infection and tissue damage and to monitor progression of chronic diseases.

Interstitial lung abnormalities have long been described based on HRCT findings, but the clinical outcome is unknown. In few cases, it may represent an early and/or mild form of pulmonary fibrosis, but in most individuals, the prognosis (progression, regression or no changes) is uncertain. However, recent evidence indicates that a significant number of them (smoker and not smokers) displays progressive imaging abnormalities over time, and that ILA are associated with a higher risk of all-cause and respiratory-cause mortality also in both smokers and non-smokers^2,14^. These findings indicate that despite often being asymptomatic, as in our cohort, the presence of ILA may be associated with progression and lower survival rates among older individuals. In this context, we evaluated the serum levels of α-Klotho in 20 individuals of this cohort, after 1-year follow-up, and again we found a positive correlation with FVC and FEV1, although DLCO did not reach statistical significance.

The pathogenic mechanisms involved in the development of ILA are unknown, but this disorder is more often found in older persons, smokers, and those having the single-nucleotide polymorphism rs35705950 in the promoter of the gene encoding mucin 5B (MUC5B)^1,2,15^.

Likely, the strongest demographic risk factor is aging although the involved mechanisms are unknown. In this context, we focus this study on α-Klotho, an anti-aging molecule that has been proved to decline with age. Furthermore, Klotho deficiency is strongly associated with human diseases related to aging such as cancer, chronic kidney disease, atherosclerosis, skin atrophy, chronic pulmonary diseases and osteoporosis^7,8,16^.

Our study demonstrated that individuals with ILA have similar serum concentrations of α-Klotho than control subjects, but importantly, supports the notion that low concentrations of this anti-aging molecule are associated with reduced lung function and could be a predictive biomarker of accelerated rate of lung function decline in individuals with ILA.

The mechanisms underlying the changes in α-Klotho in a subgroup of individuals with ILA are uncertain. It may include among others, decreased expression caused by gene polymorphims (e.g., KL-VS), promoter DNA methylation, posttranscriptional regulation by microRNAs (e.g., upregulation of miR-34a), and alterations in the shedding by ADAM10 and ADAM17 activity^17–20^. Also, expression of α-Klotho may decrease under sustained stress conditions. Interestingly, there is a bidirectional relationship between klotho and NF-κB. On one hand, klotho expression is down-regulated by a NF-κB–dependent mechanism, and on the other, klotho negatively regulates NF-κB, leading to a decrease in proinflammatory gene transduction^21–24^. A larger cohort study and basic research are needed to corroborate these results and determine the involved lung pathogenic mechanisms.

## Patients and Methods

Fifty respiratory asymptomatic adults with ILA from our ongoing Aging Lung Program (that enrolls subjects over 60 years living in Mexico City for at least 5 years), and 150 healthy individuals randomly selected from the same cohort were included in the study. Diagnosis of ILA was performed by HRCT and included mainly the presence of ground-glass abnormalities and reticulations in nondependent lung zones, with an extent above 5% of the total lung. Signed informed consent was obtained by all participants of the Program, and the study was approved by Research and Ethic Committees of our Institute (C39-14). All methods were performed in accordance with the relevant guidelines and regulations. Forced vital capacity (FVC), forced expiratory volume in 1 second (FEV_1_), diffusing capacity of the lung for carbon monoxide (DL_CO_) and six-minutes’ walk test (Oxygen desaturation and 6-MW distance) were measured at the first visit in all controls and ILA subjects. Serum levels of α-Klotho were determined by enzyme-linked immunosorbent assay (ELISA) kit (IBL America, Cat. 279988), as previously reported. C-reactive protein was measured by nephelometry. Serum concentration of MMP7 was analyzed by enzyme-linked immunosorbent assay (ELISA) according to the manufacturer’s instructions (Quantikine Human Total MMP-7 Immunoassay, R&D Systems, Inc, Minneapolis, MN).

Results were expressed as mean ± SD. Comparison between groups was performed by Fisher’s exact for categorical values and t-Test and Wilcoxon rank-sum test for continuous variables using a statistical program (Stata for Windows, version 12). The correlation between serum α-Klotho levels and the pulmonary functional tests or MMP-7 was examined by Spearman correlation test. Values of p < 0.05 were considered significant.

### Ethics approval and consent

Consent letter was signed by all participants of the Program. The study was approved by Research and Ethic Committees of Instituto Nacional de Enfermedades Respiratorias “Ismael Cosío Villegas” (C39-14). All methods were performed in accordance with the relevant guidelines and regulations.

## Data Availability

Data sharing not applicable to this article as no datasets were generated or analyzed during the current study.
